# The US21 viroporin of human cytomegalovirus stimulates cell migration and adhesion

**DOI:** 10.1128/mbio.00749-23

**Published:** 2023-07-21

**Authors:** Anna Luganini, Valentina Serra, Giorgia Scarpellino, Shree Madhu Bhat, Luca Munaron, Alessandra Fiorio Pla, Giorgio Gribaudo

**Affiliations:** 1 Department of Life Sciences and Systems Biology, University of Torino, Torino, Italy; Princeton University, Princeton, New Jersey, USA

**Keywords:** human cytomegalovirus, US21 protein, viroporin, Ca^2+^ homeostasis, cell migration

## Abstract

The human cytomegalovirus (HCMV) US12 gene family contributes to virus-host interactions by regulating the virus’ cell tropism and its evasion of host innate immune responses. US21, one of the 10 US12 genes (US12–US21), is a descendant of a captured cellular transmembrane BAX inhibitor motif-containing gene. It encodes a 7TMD endoplasmic reticulum (ER)-resident viroporin (pUS21) capable of reducing the Ca^2+^ content of ER stores, which, in turn, protects cells against apoptosis. Since regulation of Ca^2+^ homeostasis affects a broad range of cellular responses, including cell motility, we investigated whether pUS21 might also interfere with this cytobiological consequence of Ca^2+^ signaling. Indeed, deletion of the US21 gene impaired the ability of HCMV-infected cells to migrate, whereas expression of US21 protein stimulated cell migration and adhesion, as well as focal adhesion (FA) dynamics, in a way that depended on its ability to manipulate ER Ca^2+^ content. Mechanistic studies revealed pUS21-mediated cell migration to involve calpain 2 activation since its inhibition prevented the viroporin’s effects on cell motility. Pertinently, pUS21 expression stimulated a store-operated Ca^2+^ entry (SOCE) mechanism that may determine the activation of calpain 2 by promoting Ca^2+^ entry. Furthermore, pUS21 was observed to interact with talin-1, a calpain 2 substrate, and crucial protein component of FA complexes. A functional consequence of this interaction was confirmed by talin-1 knockdown, which abrogated the pUS21-mediated increase in cell migration. Together, these results indicate the US21-encoded viroporin to be a viral regulator of cell adhesion and migration in the context of HCMV infection.

**IMPORTANCE:**

Human cytomegalovirus (HCMV) is an opportunistic pathogen that owes part of its success to the capture, duplication, and tuning of cellular genes to generate modern viral proteins which promote infection and persistence in the host by interfering with many cell biochemical and physiological pathways. The US21 viral protein provides an example of this evolutionary strategy: it is a cellular-derived calcium channel that manipulates intracellular calcium homeostasis to confer edges to HCMV replication. Here, we report on the characterization of a novel function of the US21 protein as a viral regulator of cell migration and adhesion through mechanisms involving its calcium channel activity. Characterization of HCMV multifunctional regulatory proteins, like US21, supports the better understanding of viral pathogenesis and may open avenues for the design of new antiviral strategies that exploit their functions.

## INTRODUCTION

One of the most impressive features of herpesviruses is their abundance of viral proteins with clear sequence similarities to their cellular counterparts. The current belief is that, over the million years of coevolution with their hosts, herpesviruses captured many cellular genes that they then extensively optimized to encode modern viral proteins that either imitate or inhibit the original cellular function or, as in most cases, result in new functions that confer advantages to the virus (1, 2). In this regard, the human cytomegalovirus (HCMV) proves to be a master at capturing, duplicating, and shaping cellular genes to generate regulatory viral proteins able to interfere with many biochemical pathways and immune defense responses in the host, thereby promoting virus replication and persistence (3, 4). Of the numerous HCMV genes encoding proteins with signs of similarity to cellular proteins, the US12 gene family represents an astonishing example of this herpesviruses’ “capture and shape” evolutionary strategy (4, 5). The US12 gene family includes a set of 10 contiguous tandemly arranged genes (US12–US21) evolved, through multiple duplication and divergence events, from an initially host-derived gene thought to have been captured by an ancestral primate CMV (5). Low levels of amino acid sequence similarity have been observed between some of the predicted 7TMD US12 proteins and members of the transmembrane Bax-inhibitor 1 motif-containing (TMBIM) protein family (5). TMBIM are multi-transmembrane “stress sentinel” proteins that regulate multiple adaptive cellular responses to stress conditions by modulating intracellular Ca^2+^ homeostasis (6, 7). In recent years, the US12 genes have been implicated in several aspects of HCMV infection, such as the regulation of HCMV cell tropism, as observed for US16, US18, and US20 (8 - 11), the tuning of virion composition, as for US16 and US17 (9, 12), and the evasion of natural killer (NK) cell activation, as observed for US12, US14, US18, and US20 (13 - 15). However, to date, a specific biochemical function has only been defined for the US21 protein (16). In fact, pUS21 shows the highest level of identity with two cellular TMBIM members, namely, Bax-inhibitor 1 (BI-1) and Golgi anti-apoptotic protein (GAAP). Similar to these proteins, it functions as a Ca^2+^-permeable multi-transmembrane channel able to promote the release Ca^2+^ from intracellular stores, such as the endoplasmic reticulum (ER). One of the most immediate consequences of this biochemical event is the capacity of pUS21 to protect cells against apoptosis (16). pUS21 could, thus, be considered an HCMV-encoded viroporin evolved from a captured primate TMBIM homolog (16). Indeed, phylogenetic analysis has sustained the US21 gene as the initial point of entry of the US12 gene family into an ancestral primate CMV, given its location on a well-diverged branch separate from all other US12 genes (5).

The regulation of Ca^2+^ homeostasis impacts a variety of cellular functions, including energy metabolism, cell proliferation and motility, transcriptional regulation, and programmed cell death, as well as many cell responses to environmental changes and stresses (17, 18). Therefore, it is not surprising that HCMV exploits a host-derived TMBIM gene encoding a Ca^2+^ channel to modulate intracellular Ca^2+^ dynamics which, in turn, benefit its replication (19). In this regard, not only does the functional characterization of the cytobiological consequences of US21-mediated alterations to Ca^2+^ homeostasis contribute to our understanding of the virus’ pathogenetic mechanisms, but it also offers the prospect of developing new antivirals for the treatment for HCMV infection through the targeting of the HCMV-mediated dysregulation of intracellular Ca^2+^ signaling.

To fill this gap in our knowledge, we report on the functional characterization of the viroporin pUS21 as a viral regulator of cell migration and adhesion through a mechanism that depends on its Ca^2+^ channel activity, which involves both calpain activation and interaction with talin-1.

## RESULTS

### The US21 gene is involved in the migration of HCMV-infected cells

**Fig 1:**
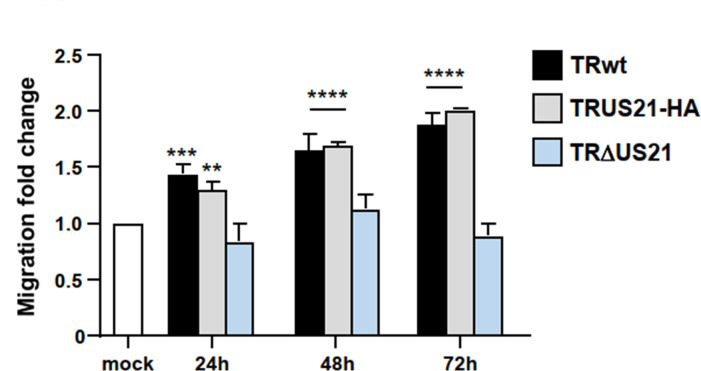
US21 is required for the migration of HCMV-infected cells**.** Serum-starved HFFs were mock-infected or infected with TRwt, TRUS21-HA, or TRΔUS21 (MOI of 1 PFU/cell) and then analyzed in chemotactic migration assays. Data shown are means ± SEM of three independent experiments performed in triplicate and analyzed using unpaired *t*-tests. *P < 0.05, **P < 0.01, ***P < 0.001, ****P < 0.0001 vs calibrator sample (mock).

Considering the central role of intracellular Ca^2+^ in the regulation of both cell adhesion and cell migration (20) and the viroporin activity of pUS21 (16), we investigated the possibility that the US21 gene plays a role in the migration of HCMV-infected cells. To this end, the migration of human foreskin fibroblasts (HFFs) infected with the low-passage TR wild-type strain (TRwt), its derivative TRΔUS21, in which the US21 ORF was deleted, or the revertant virus TRUS21-HA (16) was examined using a transwell migration assay and FBS as chemoattractant at different times post-infection (p.i.). In comparison with mock-infected cells, HFFs infected with TRwt or TRUS21-HA showed significantly more cell migration as early as 24 h p.i. (Fig. 1). By contrast, the number of migrated TRΔUS21-infected HFFs was comparable to that of mock-infected cells throughout the experiment (Fig. 1). This result demonstrated the lack of the US21 gene to impact the ability of HCMV-infected cells to migrate in response to a chemoattractant, pointing toward a role of the US21 gene in this biological consequence of HCMV infection.

### pUS21 stimulates cell migration and adhesion by regulating focal adhesion dynamics

**Fig 2:**
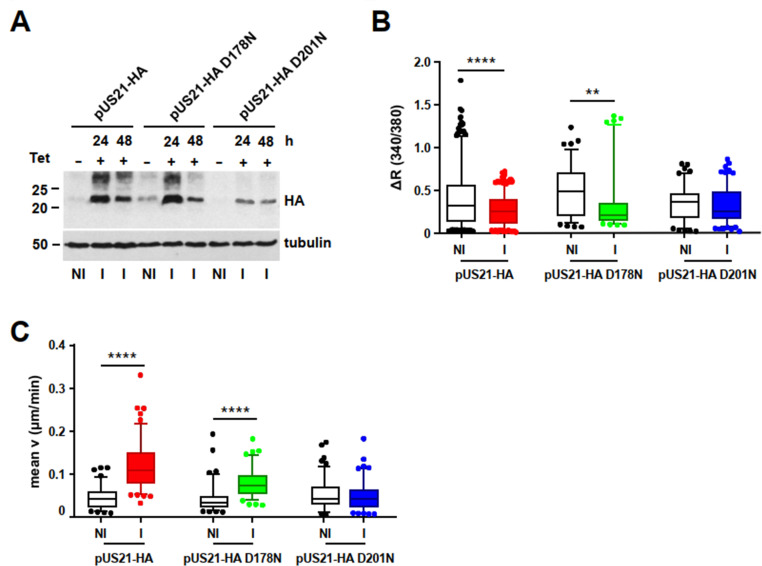
pUS21 expression stimulates cell migration. (**A**) Tetracycline-inducible expression of pUS21-HA, pUS21-HA D178N, and pUS21-HA D201N in T-REx-U2OS-derived cell lines as verified by immunoblotting with an anti-HA MAb. Protein extracts were from cells non-induced (NI) or induced (I) with tetracycline 1 µg/mL for 24 and 48 h. (**B**) Expression of pUS21 lowers the agonist-releasable Ca^2+^ content in U2OS cells. Box plot 5th–95th percentiles show the maximum cytosolic Ca^2+^ concentrations in ionomycin/thapsigargin-stimulated Fura-2 AM-loaded T-REx-U2OS cell lines. Data were analyzed by one-way ANOVA followed by the Dunn’s multiple comparison test: **P < 0.01, ****P < 0.0001; pUS21-HA NI, *n* = 380 cells; pUS21-HA I, *n* = 425 cells; pUS21-HA D178N NI, *n* = 93 cells; pUS21-HA D178N I, *n* = 87 cells; pUS21-HA D201N NI, *n* = 116 cells; and pUS21-HA D201N I, *n* = 140 cells. (**C**) Random migration of T-REx-U2OS cells induced to express pUS21-HA, pUS21-HA D178N, or pUS21-HA D201N. Data are shown as box plot 5th–95th percentiles of three independent experiments performed in triplicate. Statistical significance vs NI cells: ****P < 0.0001; pUS21-HA NI, *n* = 84 cells; pUS21-HA I, *n* = 115 cells; pUS21-HA D178N NI, *n* = 96 cells; pUS21-HA D178N I, *n* = 105 cells; pUS21-HA D201N NI, *n* = 118 cells; and pUS21-HA D201N I, *n* = 102 cells.

To investigate the mechanism through which US21 drives cell migration, a cellular system was devised for its expression in isolation in order to permit the elucidation of its specific contribution. Specifically, a tetracycline-regulated expression system (T-REx) was generated for the efficient expression of different HA-tagged US21 proteins in human U2OS cells, a cell line widely used for migration studies. In the T-REx system, the expression of pUS21 is mediated by the addition of tetracycline, thus avoiding any adverse effect on host cell physiology that may derive from the uninterrupted expression of the viroporin. T-REx-U2OS cells were engineered to express the wild-type pUS21-HA or two mutant proteins, the pUS21-HA D178N and the pUS21-HA D201N, in which the Asp178 and Asp201 residues were mutated to Asn. Asp 178 and Asp201 constitute the TMBIM family conserved pH sensor of pUS21, required for the Ca^2+^-conducting activity of pUS21 (Fig. S1) (16). The T-REx-U2OS system was validated for both the tetracycline-inducible expression of pUS21 proteins (Fig. 2A) and its Ca^2+^-mobilizing activity (Fig. 2B). In fact, as expected, the expression of pUS21-HA or pUS21-HA D178N brought about a significant reduction in the amount of Ca^2+^ releasable from intracellular stores (Fig. 2B) compared with uninduced cells or cells expressing the mutated D201N protein lacking Ca^2+^-conducting function (16). By means of time-lapse microscopy, we then evaluated whether the inducible expression of US21 proteins affected T-REx-U2OS random cell migration. As depicted in Fig. 2C, the analysis of single-cell trajectories showed the expression of pUS21-HA and pUS21-HA D178N to increase the rate of random cell migration, whereas D201N protein expression had no significant effect (Fig. 2C), thus sustaining a role of pUS21 as a functional Ca^2+^ channel in stimulating of cell migration. The same results were obtained for chemotactic migration assays (Fig. S2).

**Fig 3:**
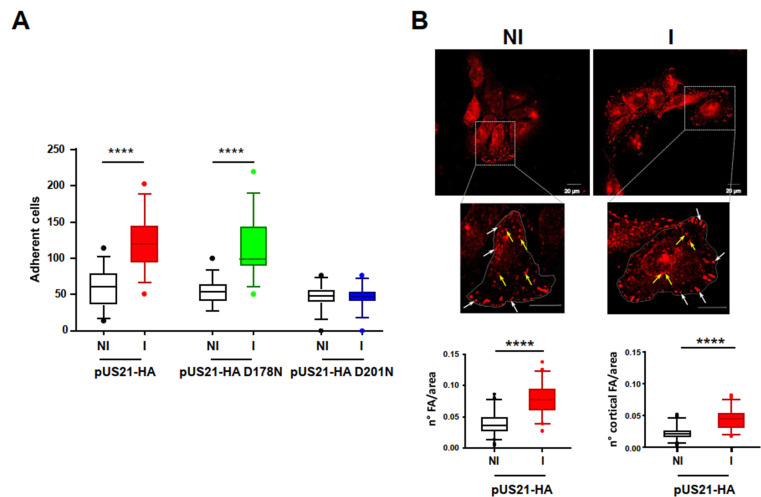
pUS21 expression enhances cell adhesion. (**A**) Adhesion of T-REx-U2OS cells that express pUS21-HA, pUS21-HA D178N, or pUS21-HA D201N. T-REx-U2OS cells were left non-induced (NI) or induced (I) with 1 µg/mL tetracycline for 48 h to express pUS21-HA, pUS21-HA D178N, or pUS21-HA D201N proteins. Thereafter, cells were detached, counted, plated, and allowed to adhere for 40 min at 37°C. Adherent cells were then stained with DAPI and microscopically counted. Results are representative of three independent experiments performed in triplicate and showed as box plot 5th–95th percentiles. Statistical significance vs NI cells: ****P < 0.0001. For all conditions, the number of microscope fields analyzed for cell counting was *n* = 32, with an average number of cells/field of pUS21-HA NI, *n* = 59 cells/field; pUS21-HA I, *n* = 123 cells/field; pUS21-HA D178N NI, *n* = 53 cells/field; pUS21-HA D178N I, *n* = 115 cells/field; pUS21-HA D201N NI, *n* = 47 cells/field; and pUS21-HA D201N I, *n* = 47 cells/field. (**B**) pUS21 expression increases the number of focal adhesions (FAs). (Upper panel) Non-induced (NI) or induced (I) for 48 h T-REx-U2OS-US21-HA cells were fixed, permeabilized, and stained for paxillin. Representative images are shown. Figures in the bottom panels present enlargements of the inset (gray box) for both NI and I representative cell images. The number of FAs was determined by counting paxillin spots on the overall cell area (lower left panel) or at the cell perimeters (cortical) only (lower right panel) of 50 cells. Data were normalized according to cell area and are shown as box plots of 5th–95th percentiles. The average of FA/area for total counts (lower left panel) for pUS21-HA NI = 0.039 ± 0.018 and for pUS21-HA I = 0.079 ± 0.024; the average of FA/area for cortical counts (lower right panel) for pUS21-HA NI = 0.022 ± 0.009 and for pUS21-HA I = 0.043 ± 0.015. Examples of cortical FAs are indicated by white arrows; yellow arrows indicate intracellular FAs. Scale bars: 20 µM. Magnification: 60×. Statistical analysis vs NI cells: ****P < 0.0001.

Given that cell migration requires the dynamic regulation of adhesion complexes, we investigated whether pUS21 influenced the ability of T-REx-U2OS cells to adhere to surfaces. As shown in Fig. 3A, the number of adherent cells was significantly increased in cultures expressing pUS21-HA or mutant D178N protein, but not in those expressing pUS21-HA D201N, pointing toward the involvement of pUS21’s viroporin activity in the regulation of cell adhesion enhancement.

Subsequently, to characterize the pUS21-mediated increase in cell adhesion further, we investigated whether its expression could control the dynamics of FAs—large multiprotein complexes linking the actin cytoskeleton to the extracellular microenvironment and which contribute to cell migration (21). Using an immunofluorescence assay, we counted the number of FAs in T-REx-U2OS cells induced, or not induced, to express pUS21-HA. Immunodetection of paxillin, a specific adaptor protein associated with FAs and involved in the regulation of cell spreading and motility (22), was used to locate the FAs. As shown in Fig. 3B, a significant increase in the number of FAs was observed in cells induced to express pUS21-HA. The FA number, normalized to the overall area of 50 cells, was significantly higher in T-REx-U2OS cells expressing pUS21-HA than in uninduced cells, irrespective of whether FAs were counted for the whole area examined (Fig. 3B, lower left) or at cell perimeter (cortical FAs) only (Fig. 3B, lower right). Thus, the increase in cell adhesion observed in pUS21-expressing U2OS cells (Fig. 3A) correlated with the increase in the number of FAs.

Taken together, these results indicate that pUS21 expression increases the rates of cell migration and adhesion and that these biological effects are derived from pUS21’s viroporin activity.

### pUS21 stimulates cell migration through the activation of calpain and SOCE

**Fig 4:**
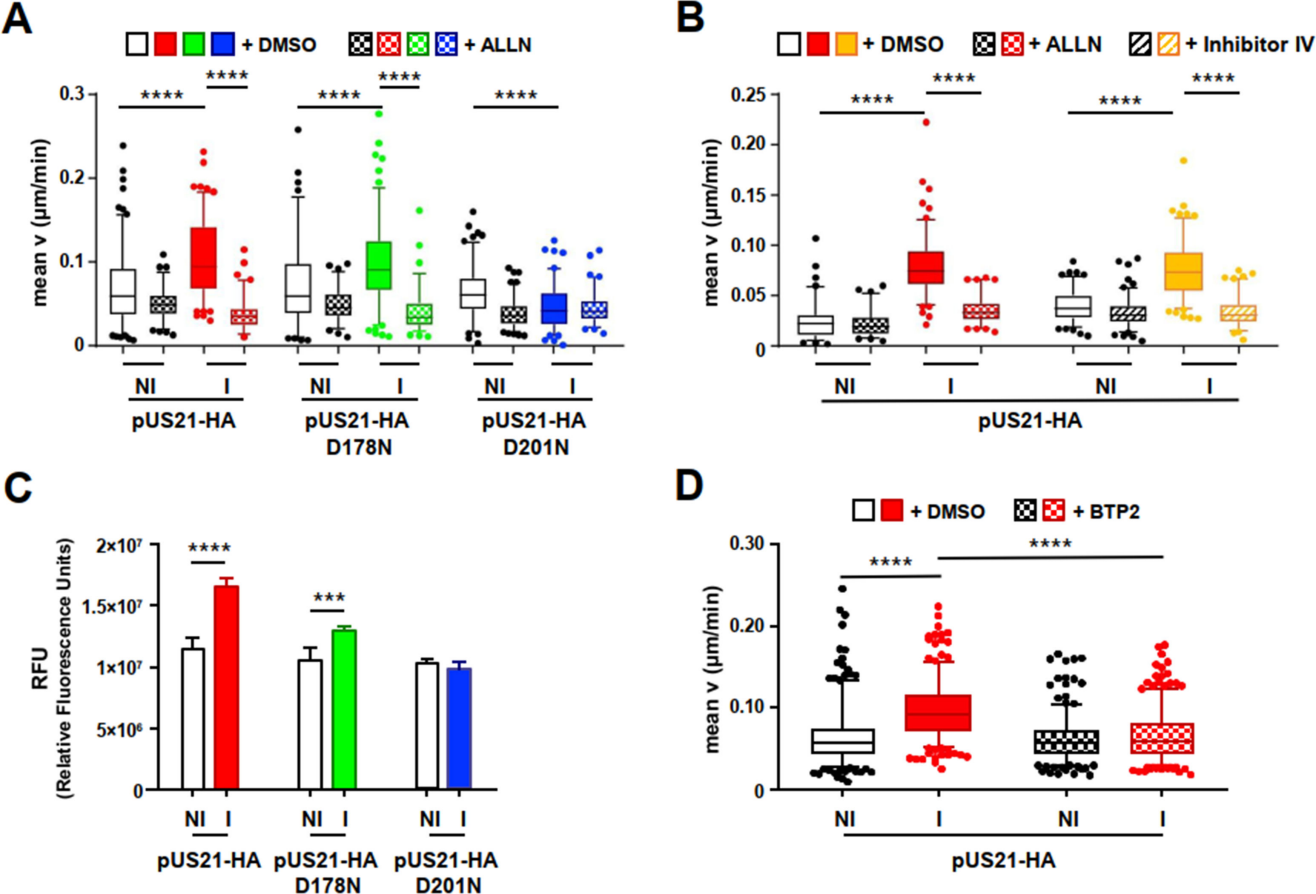
The pUS21-mediated increase in cell migration involves calpain and SOCE activation. (**A and B**) Inhibition of calpain activity abrogates pUS21-induced cell migration. T-REx-U2OS cell lines, non-induced (NI) or induced (I) for 30 h, were treated with 50 µM ALLN (calpain 1/2 inhibitor) (**A**) or inhibitor IV (calpain 2 inhibitor) (**B**) from 4 h before and during time-lapse image acquisition for random migration analysis. Images were acquired at 10 min intervals for 10 h. Box plots of 5th–95th percentiles are shown. Statistical analysis vs NI cells: ****P < 0.0001. Panel A for untreated cells: pUS21-HA NI, *n* = 140 cells; pUS21-HA I, *n* = 124 cells; pUS21-HA D178N NI, *n* = 92 cells; pUS21-HA D178N I, *n* = 138 cells; pUS21-HA D201N NI, *n* = 132 cells; pUS21-HA D201N I, *n* = 108 cells. For cells treated with ALLN: pUS21-HA NI, *n* = 98 cells; pUS21-HA I, *n* = 94 cells; pUS21-HA D178N NI, *n* = 70 cells; pUS21-HA D178N I, *n* = 76 cells; pUS21-HA D201N NI, *n* = 109 cells; pUS21-HA D201N, I *n* = 85 cells. Panel B for untreated cells: pUS21-HA NI, *n* = 116 cells (left) and *n* = 97 cells (right); pUS21-HA I, *n* = 129 cells (left) and *n* = 128 cells (right); for cells treated with ALLN: pUS21-HA NI, *n* = 131 cells and pUS21-HA I, *n* = 110 cells; for cells treated with inhibitor IV: pUS21-HA NI, *n* = 75 cells and pUS21-HA I, *n* = 82 cells. (**C**) pUS21 expression stimulates calpain activity. T-REx-U2OS-US21-HA, T-REx-U2OS-US21-HA D178N, and T-REx-U2OS-US21-HA D210N cell lines were non-induced (NI) or induced (I) with 1 µg/mL tetracycline for 48 h. Then, 200 µg of cell protein extracts was incubated with the calpain substrate (Ac-LLY-AFC) for 1 h at 37°C. Calpain enzymatic assays were determined in four independent experiments performed in triplicate. Results are expressed as means ± SEM. Statistical analysis vs NI cells: ***P < 0.001, ****P < 0.0001. (**D**) SOCE activation contributes to pUS21-stimulated cell migration. T-REx-U2OS-US21-HA cells were left non-induced (NI) or induced (I) with tetracycline for 30 h. Immediately before time-lapse image acquisition, cells were treated with 20 µM BTP2. Images were acquired at 10 min intervals for 10 h using a Nikon Plan 20× objective and a CCD camera. Data are shown as box plots of 5th–95th percentiles. Statistical analysis vs NI cells: ****P < 0.0001. For untreated cells: pUS21-HA NI, *n* = 323 cells and pUS21-HA I, *n* = 334 cells; for cells treated with BTP2: pUS21-HA NI, *n* = 304 cells and pUS21-HA I, *n* = 343 cells.

The dynamics of FAs is finely controlled by the proteolytic activity of calpain, a cellular cysteine-protease activated by local increases in cytoplasmic Ca^2+^ (23, 24). Given the Ca^2+^ leak channel activity of pUS21, we investigated whether pUS21-induced cell migration might also depend on pUS21-regulated calpain activity. First, we performed random migration assays in T-REx-U2OS cells in the presence of ALLN, a calpain 1/2 inhibitor. As shown in Fig. 4A, the addition of ALLN reduced the increase in T-REx-U2OS cell migration, induced by the expression of pUS21-HA or pUS21-D178N, down to the levels observed in uninduced cells or in cells expressing the inactive pUS21-D201N, thus supporting a role of calpain 1/2 in pUS21-induced cell migration. Next, since the activity of calpain 2 is required for efficient disassembly of FAs (25), random migration assays were repeated in the presence of a specific inhibitor of calpain 2, namely, inhibitor IV (26). As depicted in Fig. 4B, inhibitor IV prevented the pUS21-HA-induced increase in T-REx-U2OS cell migration, confirming the requirement of calpain 2 activity for pUS21-stimulated cell migration. Neither ALLN nor inhibitor IV affected the levels of tetracycline-stimulated pUS21-HA expression (Fig. S3).

Then, to sustain further involvement of calpain activation, its enzymatic activity was measured by means of fluorometric assay in extracts from T-REx-U2OS expressing the different pUS21 proteins. The expression of US21 or D178N proteins significantly increased proteolytic calpain activity compared with non-induced cells or T-REx-U2OS cells expressing the inactive D201N protein (Fig. 4C).

On the whole, these results indicate that the proteolytic activity of calpain 2 is required for pUS21-mediated regulation of cell motility and that the Ca^2+^-signaling activity of pUS21 is needed for the activation of calpain’s enzymatic activity.

However, the release of Ca^2+^ from ER through pUS21’s viroporin activity could lead to a Ca^2+^-dependent activation of calpain 2 through a store-operated Ca^2+^ entry (SOCE) mechanism. Indeed, SOCE allows extracellular Ca^2+^ to enter cells and replenish the intracellular stores following their depletion due to the activity of Ca^2+^ channels located in the ER (27). Thus, SOCE might be activated by pUS21-dependent ER Ca^2+^ depletion. To address this question experimentally, random cell migration assays were performed in the presence of BTP2, a specific inhibitor of SOCE (28) that did not alter the expression of pUS21-HA in induced T-REx-U2OS-US21-HA cells (Fig. S3). BTP2 was effective as a SOCE inhibitor in these US21-HA expressing cells (Fig. S4) and significantly reduced their migration (Fig. 4D). This result, therefore, supports the hypothesis that pUS21 leads to SOCE activation, which may, in turn, contribute to calpain activation.

### The interaction with talin-1 is required for the pUS21-mediated increase in cell migration

**Fig 5:**
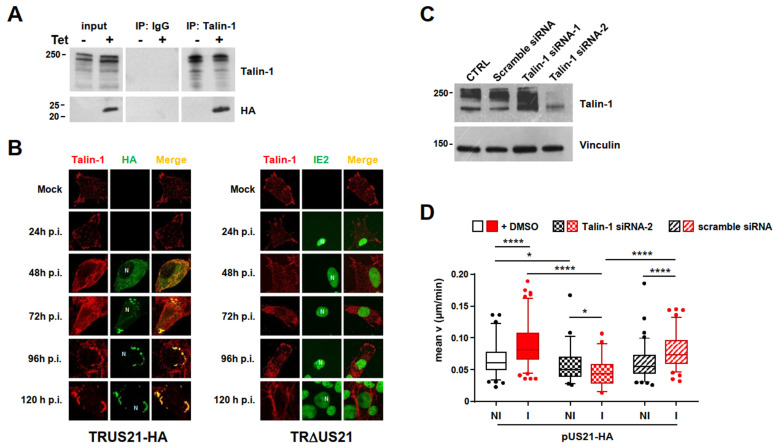
The interaction of pUS21 with talin-1 is required for the pUS21-dependent stimulation of cell migration. (**A**) Interaction of pUS21 and talin-1 in T-REx-U2OS-US21-HA cells. Cell protein extracts were prepared from cells non-induced (NI) or induced (I) for 48 h and immunoprecipitated using anti-talin-1 MAb or a non-specific control mouse IgG. Then, immunoprecipitates and input cell extracts were analyzed by immunoblotting to detect talin-1 and HA epitopes. (**B**) pUS21 and talin-1 colocalize in infected HFFs. HFFs were infected with TRUS21-HA (left), TRΔUS21 (right) (MOI of 1 PFU/cell), or mock infected. At various times p.i., cells were fixed, permeabilized, and immunostained with anti-HA (green) and an anti-talin-1 (red) MAbs for TRUS21-HA-infected cells or with an anti-talin-1 (red) MAb and anti-IE2 (green) PAb for TRΔUS21-infected HFFs. The location of nuclei is indicated by N. Magnification: 63×. (**C**) Silencing of talin-1 protein expression by siRNAs. T-REx-U2OS-US21-HA cells were transiently transfected with 100 nM of 27-nucleotide oligo duplexes human talin-1 specific sequence (siRNA-1 and siRNA-2), or a scrambled siRNA control (a negative control for specific gene downregulation), or not transfected (CTRL). After 96 h, total cell extracts were prepared and analyzed by immunoblotting with mouse anti-talin-1 MAb and vinculin (control for protein loading). (**D**) Talin-1 expression is required for pUS21-mediated increase in cell migration. T-REx-U2OS-US21-HA cells were transiently transfected with 100 nM of human talin-1 siRNA-2 or scrambled siRNA. After 48 h, cells were left non-induced (NI) or induced (I) with tetracycline for 30 h before time-lapse image acquisition for random migration analysis. Images were acquired at 10 min intervals for 10 h. Data are displayed as box plots of 5th–95th percentiles for three independent experiments performed in triplicate. *P < 0.05, ****P < 0.0001. For non-transfected cells: pUS21-HA NI, *n* = 93 cells and pUS21-HA I, *n* = 115 cells; for cells transfected with talin-1 siRNA-2: pUS21-HA NI, *n* = 46 cells and pUS21-HA I, *n* = 54 cells; for cells transfected with scramble siRNA: pUS21-HA NI, *n* = 92 cells and pUS21-HA I, *n* = 87 cells.

To gain further insight into the mechanisms underlying the regulation of cell migration by pUS21, pUS21-interacting cellular partners were identified by mass spectrometry analysis of pUS21-HA-coimmunoprecipitated complexes from non-induced or induced T-REx-U2OS cells. Among the cellular proteins co-immunoprecipitated by an anti-HA MAb in US21-HA-expressing cells, talin-1 was identified as the most probable cellular partner of pUS21 (Table S1). Talin-1 is a FA protein playing a central role in cell adhesion by regulating the activation of integrins and their linking to the actin cytoskeleton (29, 30). The interaction between pUS21 and talin-1 was confirmed by co-immunoprecipitation (Fig. 5A). Moreover, co-localization of the two proteins was observed in immunofluorescence analyses of HFFs infected with TRUS21-HA; specifically, talin-1 accumulated in cytoplasmic peripheral vesicles at late times p.i., with a staining pattern that overlapped with pUS21 (Fig. 5B, left panel). By contrast, in HFFs infected with the US21-deficient virus (TRΔUS21; Fig. 5B, right panel), the intracellular location of talin-1 was similar to that in mock-infected cells, with a staining pattern that located the protein near to the cell perimeter, as expected, rather than in peripheral cytoplasmic structures as observed in TRUS21-HA-infected cells.

The importance of the talin-1-pUS21 interaction in the context of pUS21-mediated cell migration was then investigated using random migration assays (Fig. 5D) of T-REx-U2OS-US21-HA cells in which the expression of talin-1 was knocked down by siRNA (Fig. 5C). The expression of a scrambled siRNA did not affect the pUS21-HA-dependent enhancement of U2OS cell migration (Fig. 5D). However, the migration speed of cells expressing pUS21-HA, but in which talin-1 expression was suppressed, was significantly lower than that of induced cells transfected with scramble siRNA (Fig. 5D), suggesting that the interaction between talin-1 and pUS21 was required for the pUS21-dependent stimulation of cell migration.

## DISCUSSION

**Fig 6:**
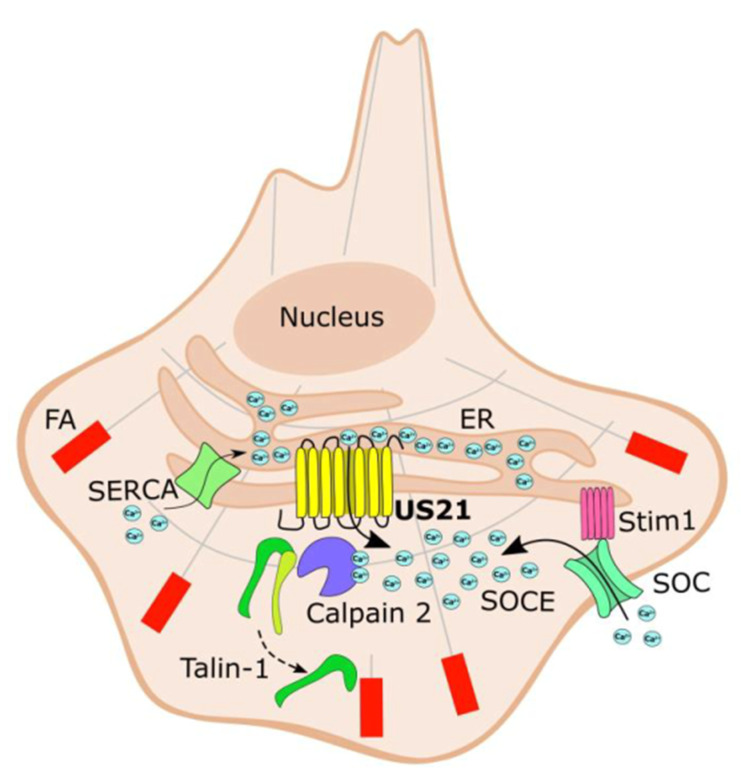
Control of cell adhesion and migration by the US21 viroporin of HCMV. The channel function of pUS21 leads to depletion of ER Ca^2+^ content, which is then detected by the Stim1 sensor that, in turn, triggers the activation of SOCE and the influx of Ca^2+^ from the extracellular environment through SOC channels. The resulting increase in cytoplasmic Ca^2+^ activates calpain-2, talin-1 proteolysis, and the turnover of focal adhesions. Overall, this pUS21-dependent mechanism stimulates cell spread and increases the speed of cell migration. FA, focal adhesion; SERCA, sarco/endoplasmic reticulum Ca^2+^ ATPase pump; SOC, store-operated Ca^2+^ channel; SOCE, store-operated Ca^2+^ entry mechanism; Stim1, stromal interaction molecule 1 that functions as an ER Ca^2+^ sensor.

This study provides evidence that enables us to model the role of HCMV US21 protein in regulating cell migration via a mechanism that depends on its Ca^2+^ channel activity (Fig. 6). The model predicts the activation of a SOCE mechanism following the pUS21-mediated depletion of ER Ca^2+^ stores. Store-operated calcium entry usually follows the detection of a decrease in luminal Ca^2+^ content via the ER Ca^2+^ sensor STIM1, which then interacts with and activates plasma membrane store-operated Ca^2+^ channels (SOCs), stimulating the Ca^2+^ influx from the extracellular environment to replenish the depleted intracellular ER store (27). The resulting elevated cytosolic Ca^2+^ concentration may consequently stimulate calpain 2 activation near focal adhesions, thus leading to an increase in their turnover and enhancing cell migration (Fig. 6).

An interesting observation was the requirement for the US21 gene for the migration of HCMV-infected cells (Fig. 1). Previous findings had associated HCMV-stimulated cell motility and adhesion to the activity of the virus-encoded G protein-coupled receptor US28, which, upon binding to CC chemokines, induces smooth muscle cell and myeloid cell migration, as well as monocytes adhesion to endothelial cells (31, 32). These US28-dependent effects on cell motility have been suggested to impact HCMV infection *in vivo* by promoting viral dissemination and contributing to both the development of HCMV-mediated vascular diseases and the virus’ oncomodulatory role (31 - 34). However, the migration of HCMV-infected cells has also been described in epithelial cell systems, even in the absence of exogenous chemokine stimulation (35), meaning that US28-independent mechanisms of HCMV-induced cell migration must also exist. Considering the findings reported herein, we can postulate that the US21 viroporin contributes to the overall protein toolbox evolved by HCMV to promote the motility of infected cells and viral spread within the host.

A second point worth discussing concerns the proposed mechanism of US21-dependent cell migration (Fig. 6). Astonishingly, an almost perfect superimposable mechanism has already been described for the humanTMBIM4 (hGAAP), for the control of cell motility through SOCE and calpain 2 activation (36). hGAAP is the cellular protein that pUS21 most closely resembles, with 21% amino acid identity (AAI); indeed, the modern US21 gene could be considered a descendant of a captured primate TMBIM4 ancestor gene (16). Interestingly, viral orthologs of hGAAP have been found in some orthopoxviruses, such as camelpox (CMLV), cowpox, and some strains of VACV (37, 38). These viral GAAPs share 73% AAI with hGAAP (37, 38), favoring the possibility that the TMBIM4 gene was acquired more recently by an ancestral poxvirus compared to the acquisition of a remote precursor of the US21 gene by an ancient primate CMV (16). Nonetheless, and despite the low level of conservation between pUS21 and CMLV GAAP (vGAAP) (20% AAI), both viral proteins form Ca^2+^ channels, protect against apoptotic stimuli, and increase cell migration (16, 39). Indeed, the critical amino acid residues required for regulating the Ca^2+^-conducting activity of BsYetJ—a bacterial BI-1 homolog from *Bacillus subtilis* and the structural model for mammalian TMBIM proteins (7, 40)—can be found in both pUS21 and vGAAP. The presence of the two TMBIM family-conserved C-terminal aspartic acid residues (Asp-178 and Asp-201 in pUS21; Asp-196 and Asp-219 in vGAAP), which may form hydrogen-bonded salt bridges between each other and with the basic Arg residue on TMD2 (Arg-69 in pUS21 and Arg-90 in vGAAP), in fact, sustains the view that the Ca^2+^ viroporin activity of both vGAAP and pUS21 constitutes their core function and gives rise to the subsequent cytobiological consequences of these proteins, such as protection from apoptosis and the stimulation of cell motility (38).

It must be emphasized, however, that the manipulation of cellular Ca^2+^ homeostasis by both vGAAP and pUS21 represents a clear instance of a convergently evolved mechanism attained by two unrelated viral pathogens, which independently captured and shaped a cellular TMBIM4 ancestral gene to derive regulatory viral proteins with superimposable functions in regulating virus-host cell interactions.

## MATERIALS AND METHODS

### Bioinformatics and protein modeling

The Phyre2 server (41) was used for fold recognition-based modeling of pUS21. The crystal structures of BsYetJ in the open (Protein Data Bank [PDB] ID code 4PGS) and closed (PDB ID code 4PGR) conformations (40) were used as templates to generate models of pUS21 as previously described (16). Molecular graphics of the pUS21 models were generated using the UCSF Chimera package.

### Compounds

ALLN inhibitor (A6185, calpain 1/2 inhibitor), inhibitor IV (208724, calpain 2 inhibitor), and BTP2 (203890M, CRAC channel inhibitor) were purchased from Sigma-Aldrich and resuspended in DMSO.

### Cells and viruses

hTERT-immortalized HFFs and the human osteosarcoma U2OS cell line (ATCC HTB-96) were grown in DMEM (Euroclone) supplemented with 10% tetracycline-reduced FBS (Euroclone). HCMV TR (42) and its derivatives TRΔUS21 and TRUS21-HA were reconstituted and titrated as previously described (16).

### US21 protein expression

The tetracycline (Tet)-induced expression of pUS21-HA, pUS21-HA D178N, and pUS21-HA D201N was attained using the T-REx system (Invitrogen, Waltham, MS, USA, and Life Technologies, Waltham, MA, USA). To this end, U2OS cells were co-transfected with pcDNA6/TR plasmid (Invitrogen, ) carrying the *Tet repressor* gene and pcDNA4/TO plasmids (Invitrogen, Waltham, MA, USA) containing the different US21-HA ORFs (16). Stably transfected T-REx-U2OS cell lines were selected using 2 µg/mL blasticidin and 500 µg/mL zeocin (Life Technologies, Carlsbad, CA, USA). To induce the expression of pUS21-HA proteins, tetracycline (1 µg/mL) was added for 24 or 48 h.

### Cytosolic Ca^2+^ measurement

Cytosolic calcium quantification was determined as previously described (16, 43). Briefly, non-induced (NI) or induced (I) T-REx-U2OS cell lines, seeded at a density of 5,000 cells/cm^2^ on glass coverslips, were loaded (30 min at 37°C) with 2 µM Fura-2 AM (Invitrogen, Waltham, MA, USA) and excited at two alternating frequencies: 340 and 380 nm. The emitted fluorescence was captured using a Nikon Eclipse TE-2000S inverted microscope equipped with the MetaFluor Imaging System (Molecular Devices, San Jose, CA, USA). Ratiometric cytosolic Ca^2+^ ([Ca^2+^]_c_) measurements were expressed as the ratio (R) of fluorescence emitted at 510 nm for the two excitation wavelengths. For each condition, at least 50 regions of interest were selected, each corresponding to a single cell in the chosen image field. Images were acquired every 3 s. Ca^2+^ imaging analysis was performed by peak amplitude quantification using Clampfit 11.1 (Axon pClamp, Molecular Devices). Only the responses with a ΔR340/380 > 0.05 were considered.

To assess the ER Ca^2+^ content in T-REx-U2OS NI or I cells, depletion of the ER Ca^2+^ pool was triggered using Thapsigargin (TG, 2 µM) plus Ionomycin (IONO, 5 µM), in the absence of extracellular Ca^2+^ (0 Ca^2+^), and compared with subsequent increases in [Ca^2+^]_c_. Store-operated Ca^2+^ entry (SOCE) was evaluated using the “Ca^2+^ add-back” protocol. Briefly, cells were treated with TG (2 µM) in Ca^2+^-free medium to induce the depletion of Ca^2+^ stores (0 Ca^2+^out; tyrode solution without CaCl_2_ added with 2 mM ethylene glycol tetraacetic acid, EGTA). Ca^2+^-containing solution (2 mM Ca^2+^) was then added to the extracellular environment so that SOCE could be measured.

### Migration assays

Chemotactic migration assays were performed with HFFs incubated in DMEM-0.5% FBS for 24 h before infection. Then, HFFs were mock-infected or infected with TRwt, TRUS21-HA, or TRΔUS21 at an MOI of 1. Infected cells were trypsinized at 24, 48, and 72 h p.i., collected in serum-free medium, and added to the upper side of 8 µm PET 24-well multiwell inserts system (BD, Franklin Lakes, NJ, USA, and Falcon, Glendale, AZ, USA) at a density of 7 × 10^4^ cells per insert. The lower chamber was filled with DMEM supplemented with 10% FBS. The cells were allowed to migrate through the PET membrane for 24 h at 37°C in 5% CO_2_. Each condition was performed in triplicate. Non-migrating cells were removed from the upper side of the inserts with a cotton swab. The cells which had migrated to the underside of the inserts were fixed with cold 100% methanol and stained with 0.1% crystal violet for 30 min at room temperature (RT). The number of cells was counted for at least 15 different fields on an Olympus IX50 fluorescence microscope equipped with Image-Pro Plus software. Chemotactic migration assays of T-REx-U2OS-US21-HA, T-REx-U2OS-US21-HA D178N, and T-REx-U2OS-US21-HA D210N cells uninduced (NI) or induced (I) for 48 h with 1 µg/mL tetracycline (Tet) were carried out as described above.

For the random migration assay, T-REx-U2OS-US21-HA, T-REx-U2OS-US21-HA D178N, or T-REx-U2OS-US21-HA D210N cell lines were seeded at low density (4,000 cells/well) on fibronectin-coated 24-well plates. On the following day, cells were left uninduced or induced with 1 µg/mL tetracycline for 30 h before image acquisition for random migration or immediately subjected to time-lapse image acquisition. To assess the effect of calpain 1/2 or calpain 2 inhibition, T-REx-U2OS cell lines were incubated with 50 µM of ALLN or inhibitor IV, 4 h before and then throughout time-lapse acquisition. In all experiments, control cells were treated by adding DMSO. To verify SOCE activation in T-REx-U2OS-US21-HA cells, they were treated with 20 µM BTP2 immediately before time-lapse and throughout imaging acquisition. To test the effects of talin-1 gene knockdown on cell migration, T-REx-U2OS-US21-HA cells were transfected with talin-1 siRNA-2 or scramble siRNA as a control. After 24 h, transfected cells were detached and plated for the random migration assay as described above. In all time-lapse experiments, cells were imaged using a Nikon Eclipse Ti inverted microscope equipped with a A.S.I. MS-2000 stage and an OkoLab incubator (to keep cells at 37°C and 5% CO_2_). Images were acquired at 10 min intervals for 10 h using a Nikon Plan 20× objective and a CCD camera. MetaMorph software (Molecular Devices, San Jose, CA, USA) was used for image acquisition. Migration tracks of individual cells were generated using the ImageJ Manual Tracking plugin.

### Adhesion assay

T-REx-U2OS-US21-HA cells were seeded on 1% gelatin-coated 12-well plates. The following day, cells were left uninduced (NI) or induced (I) with 1 µg/mL tetracycline for 30 h. Cells were then detached, counted carefully, and seeded on 96-well plates (3,000 cells/well) that had been coated with 1% gelatin for 60 s. Plates were incubated for 40 min at 37°C, the medium was discarded, and then, cells gently washed three times with warm PBS 1× to remove all non-adherent cells. Fixation of adherent cells was performed at room temperature by adding 1% paraformaldehyde (Sigma-Aldrich, St. Louis, MO, USA) for 15 min. Nuclei of fixed cells were then stained with DAPI after permeabilization with 0.1% Triton X-100 in PBS 1× for 10 min at 4°C, and then 2 µg/mL DAPI in PBS 1× was added for 5 min at 37°C. Images of adherent cells were acquired using a Nikon Eclipse Ti inverted microscope equipped with a A.S.I. MS-2000 stage and an OkoLab incubator (to keep cells at 37°C and 5% CO2). MetaMorph software was used for image acquisition, and cell counting was performed by ImageJ Automated Count software.

### Calpain activity measurement

Calpain activity was measured using the Calpain Activity Fluorometric Assay Kit (Merck, Darmstadt, Germany). Fluorescence was detected using a FilterMax F5 multi-mode microplate reader.

### Talin-1 knockdown

T-REx-U2OS-US21-HA cells were transfected with 100 nM talin-1 siRNA oligonucleotide duplexes (SR304853, Origene) using the siTran2.0 siRNA transfection reagent (Origene, Rockville, MD, USA).

### Analysis of proteins

For the immunofluorescence analysis of focal adhesions (FAs), T-REx-U2OS-US21-HA cells were induced with 1 µg/mL tetracycline for 48 h. Then, cells were fixed in 1% paraformaldehyde (15 min, RT), permeabilized with 0.2% Triton X-100 in blocking buffer (10% FBS and 5% glycine in PBS 1×) (20 min, 4°C), and incubated with rabbit MAb anti-paxillin (clone 04-581, Millipore, Burlington, MA, USA) (1:200) for 2 h at 37°C followed by a 568-conjugated donkey anti-rabbit IgG antibody (Life Technologies, Carlsbad, CA, USA) for 1 h at 37°C. FAs were then visualized using a Nikon Eclipse Ti fluorescence microscope equipped with Oculus software and counted using the ImageJ Manual Cell Counting plugin.

Immunofluorescence analysis of TRUS21-HA or TRΔUS21 infected HFF cells was performed as previously described (16, 44, 45) using the rat MAb anti-HA (clone 3F10, Roche) (1:50), the homemade rabbit PAb anti-IE2 (1:450), and the mouse MAb anti-talin-1 (clone 8d4, Sigma-Aldrich) (1:100). The binding of primary antibodies was detected by 488-conjugated goat anti-rat IgG (Sigma-Aldrich), 488-conjugated goat anti-rabbit IgG (Life Technologies, Carlsbad, CA, USA), and 594-conjugated rabbit anti-mouse IgG antibodies (Sigma-Aldrich), respectively. Cells were then visualized with a Leica TCS SP5 multiphoton-inverted confocal microscope equipped with LAS AF matrix software.

Immunoblotting was performed as previously described (9, 11, 16). Proteins were immunostained using a rat anti-HA MAb (clone 3F10, Roche, 1:100), a mouse MAb anti-talin-1 (clone 8d4, Sigma-Aldrich), a mouse MAb anti-tubulin (clone TUB 2.1, Sigma-Aldrich), or a mouse anti-vinculin MAb (clone V9264, Sigma-Aldrich). The last two were used as a control for cellular protein loading.

For co-immunoprecipitation (co-IP), T-REx-U2OS-US21-HA cells were induced for 48 h with 1 µg/mL tetracycline or left non-induced as a control. Cytoplasmic proteins were extracted in Triton buffer [20 mM Tris-Cl, pH 6.8, 100 mM NaCl, 1% Triton X-100, 25 µl/mL protease inhibitor cocktail (P8340, Sigma-Aldrich)], as previously described (9). For co-IP, aliquots of 100 µL of Protein A/G-agarose beads (Thermo Scientific, Waltham, MA, USA) were incubated at 4°C overnight with rotation in the presence of 10 µL of mouse MAb anti-talin-1 or control normal mouse IgG (Sigma-Aldrich). Beads were then pelleted and washed three times with Triton buffer and incubated with 500 µg of cytoplasmic protein extracts at 4°C overnight with rotation. The following day, beads were pelleted and washed three times with Triton buffer and denatured in Laemmli sample buffer 1× at 95°C for 5 min. After centrifugation, supernatants were analyzed by immunoblotting with anti-HA and talin-1 MAbs as described above.

For proteomic analysis, T-REx-U2OS-US21-HA cells were left non-induced (NI) or induced (I) for 48 h with 1 µg/mL tetracycline, and cytoplasmic cell proteins were extracted as described above. Then, aliquots of 10 mg of protein extracts from NI or I cells were immunoprecipitated using the anti-HA affinity matrix (clone 3F10, Sigma Aldrich). Immunoprecipitates were eluted, protein complexes were subjected to proteolytic digestion, and peptide mixtures were analyzed by mass spectrometry at Ion Source & Biotechnologies S.r.l. (Milan) as previously described (46). Mass spectra were obtained using a q-TOF mass analyzer (Bruker Daltonics, Billerica, MA, USA). LC/SACI and LC/micro-ESI mass chromatograms were obtained using both full-scan (MS) and tandem mass spectrometry (MS/MS) modes. The LC/SACI-MS peptide profiles of digested proteins were compared with protein and peptide sequences of a generic database using the MS-BLAST algorithm. Only interactions observed specifically in induced immunoprecipitates were considered for statistical analysis.

### Statistical analysis

All statistical analyses were performed using GraphPad Prism version 8.00 (GraphPad Software). Data are presented as the means ± SEM, and statistical analysis was performed by the Mann–Whitney test or the one-way ANOVA test. *P* values ≤ 0.05 were considered significant.

## SUPPLEMENTAL MATERIAL

Figure S1 mbio.00749-23-s0001.tif Structural models of pUS21.

Figure S2 mbio.00749-23-s0002.tif Migration of T-REx-U2OS cells expressing pUS21-HA proteins.

Figure S3 mbio.00749-23-s0003.tif Neither calpain inhibitors nor BTP2 affect the tetracycline-induced expression of pUS21-HA.

Figure S4 mbio.00749-23-s0004.tif BTP2 abolished thapsigargin-induced SOCE but not Ca^2+^ release from the ER.

Table S1 mbio.00749-23-s0005.docx Main pUS21 cellular protein partners in T-REx-U2OS cells expressing pUS21-HA.

ASM does not own the copyrights to Supplemental Material that may be linked to, or accessed through, an article. The authors have granted ASM a non-exclusive, world-wide license to publish the Supplemental Material files. Please contact the corresponding author directly for reuse.
