# Supplementary figures and images for: The US21 viroporin of human cytomegalovirus stimulates cell migration and adhesion
Source: mBio. 2023 Jul 21;14(4):e00749-23. doi: 10.1128/mbio.00749-23 (PMC10470750; doi:10.1128/mbio.00749-23)

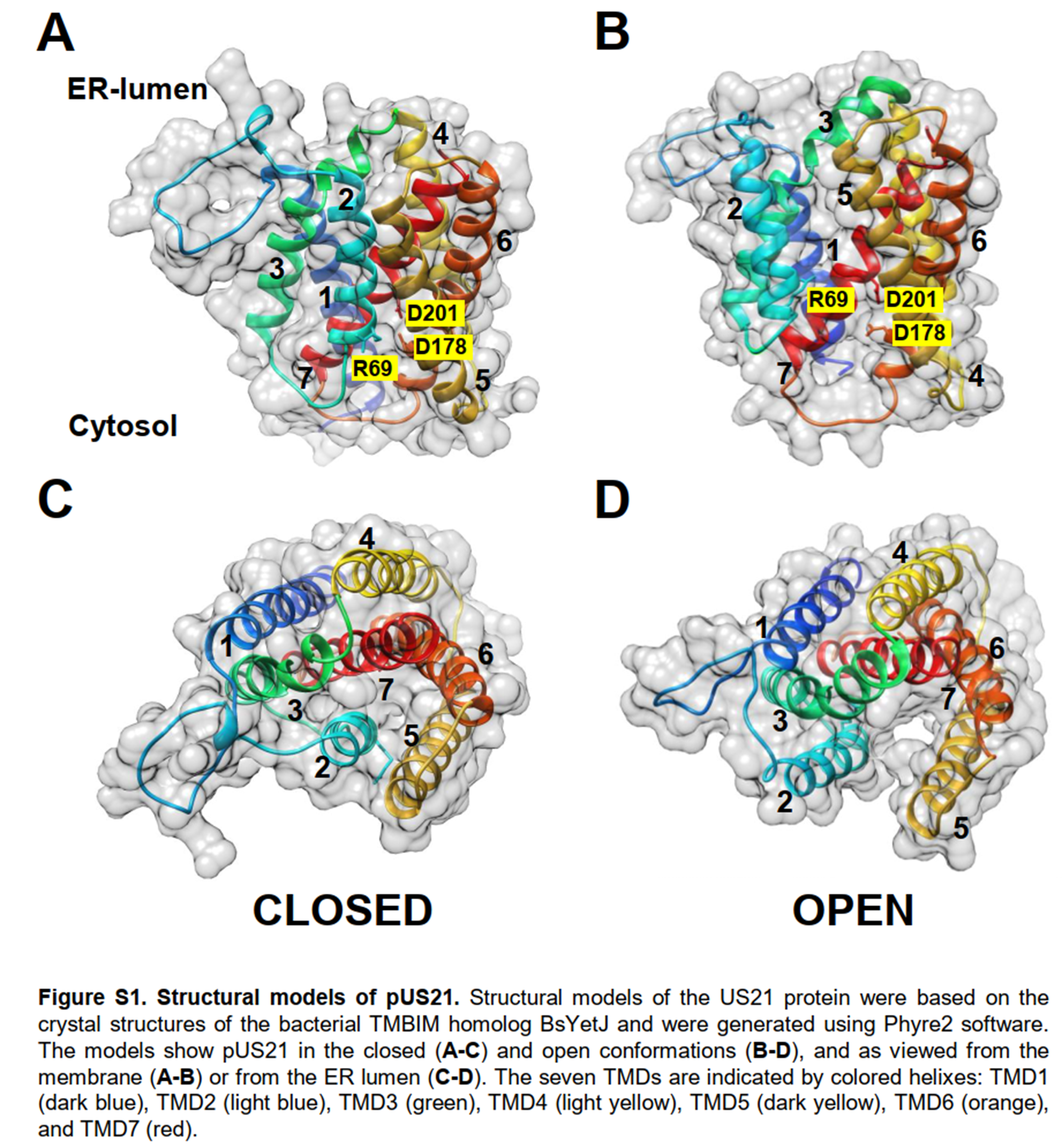

Supplement: Figure S1 — Structural models of pUS21. [file mbio.00749-23-s0001.tif]

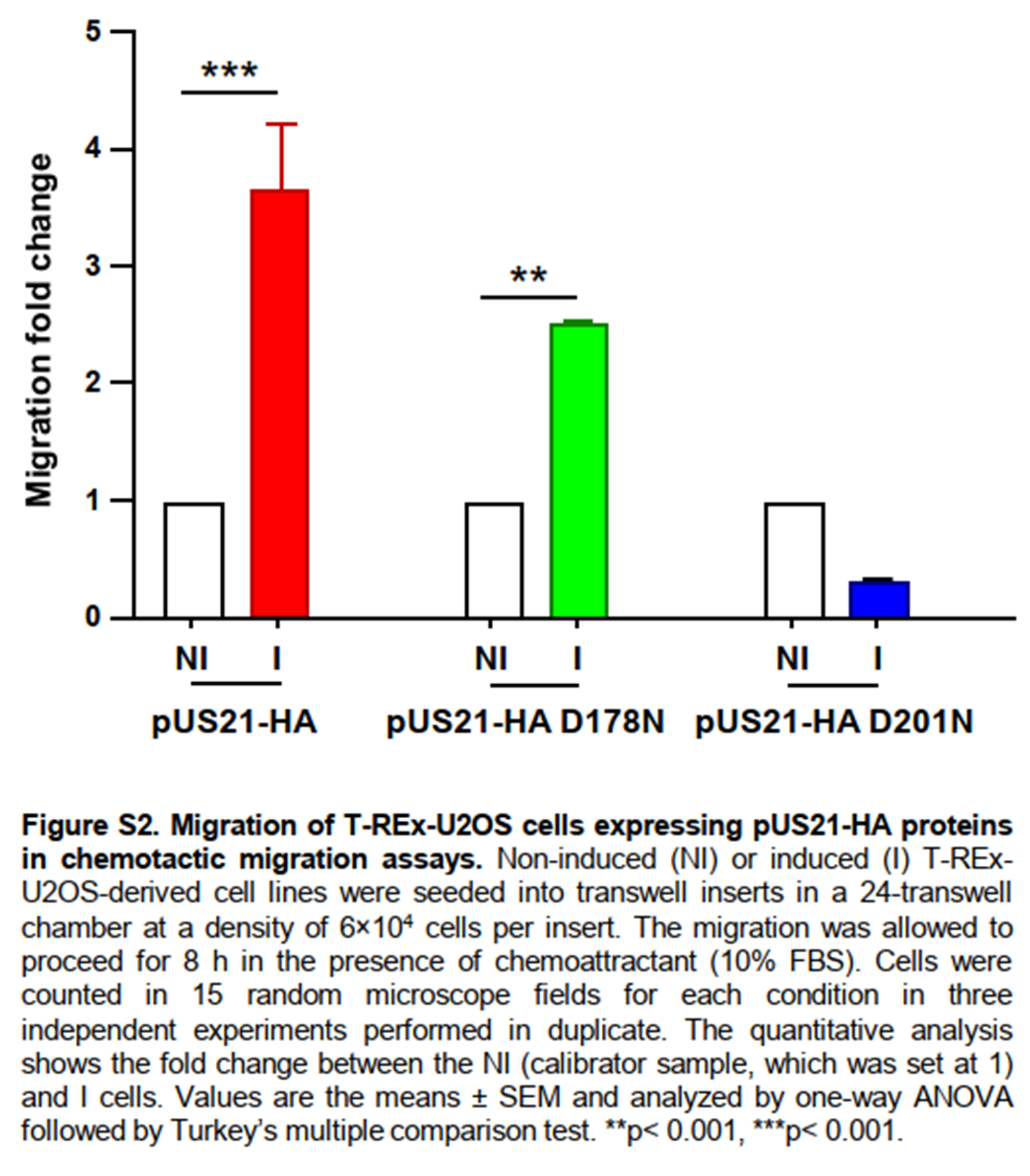

Supplement: Figure S2 — Migration of T-REx-U2OS cells expressing pUS21-HA proteins. [file mbio.00749-23-s0002.tif]

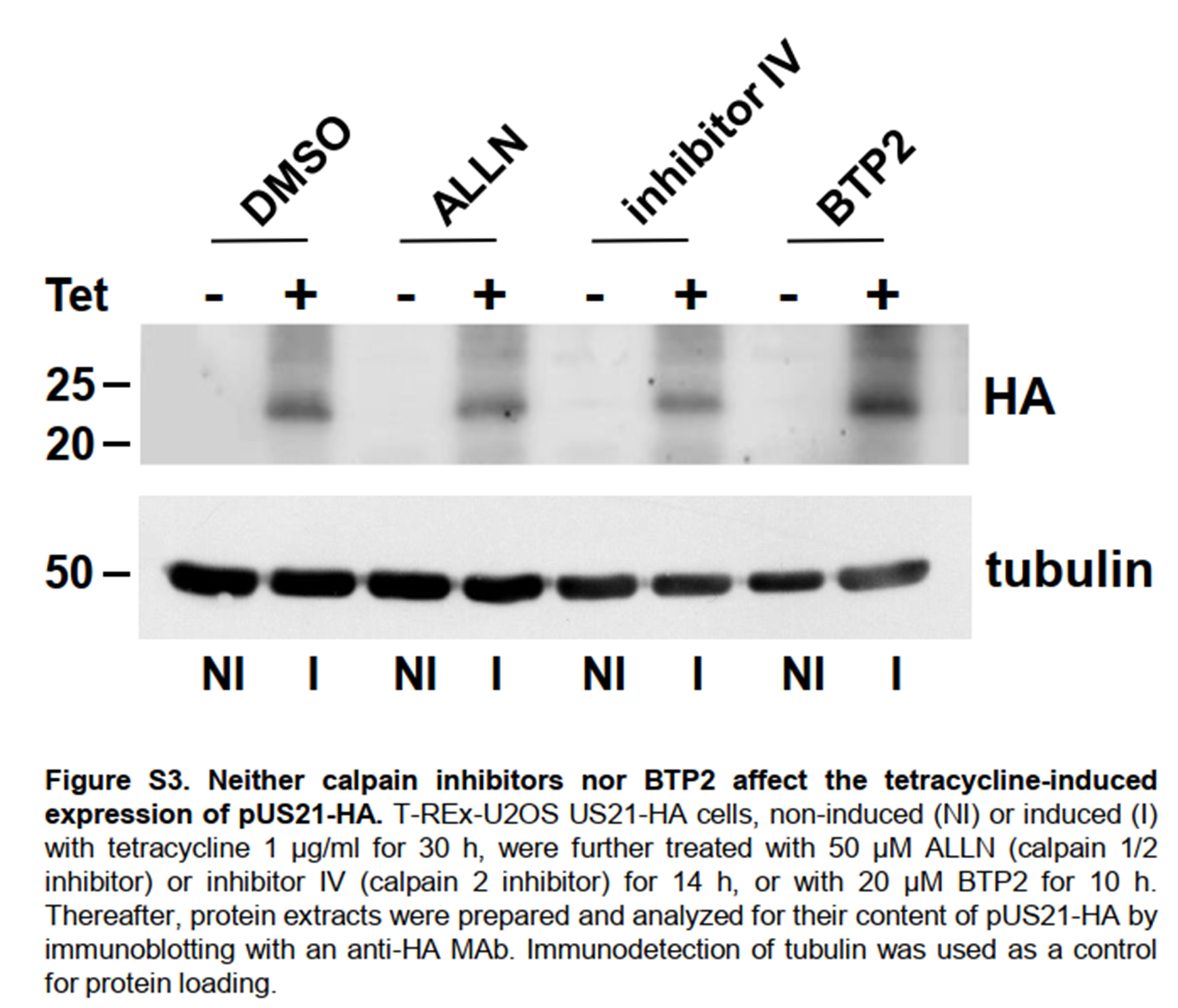

Supplement: Figure S3 — Neither calpain inhibitors nor BTP2 affect the tetracycline-induced expression of pUS21-HA. [file mbio.00749-23-s0003.tif]

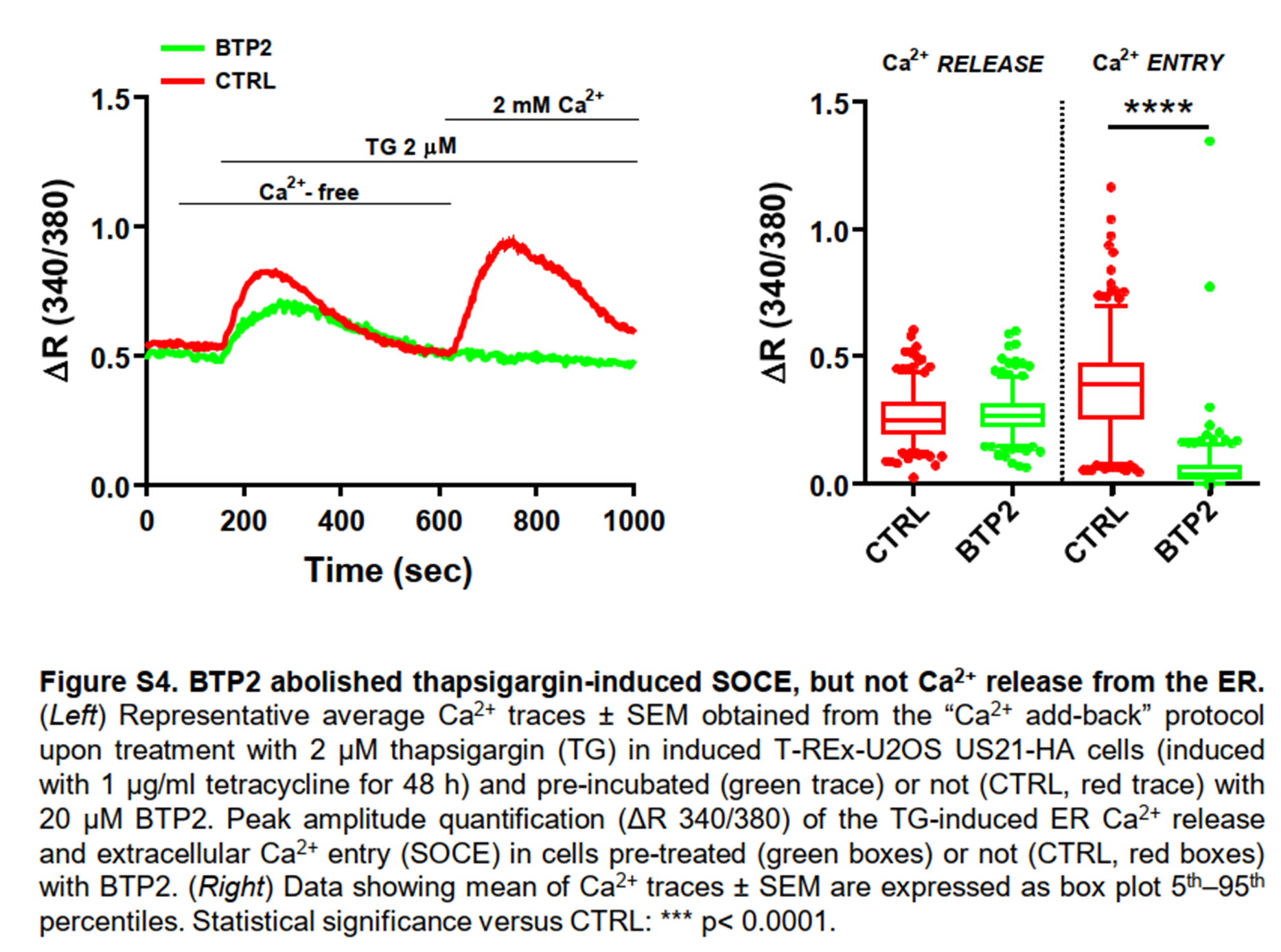

Supplement: Figure S4 — BTP2 abolished thapsigargin-induced SOCE but not Ca2+ release from the ER. [file mbio.00749-23-s0004.tif]
