# Supplementary material for: The US21 viroporin of human cytomegalovirus stimulates cell migration and adhesion
Source: mBio. 2023 Jul 21;14(4):e00749-23. doi: 10.1128/mbio.00749-23 (PMC10470750; doi:10.1128/mbio.00749-23)
Supplement: Table S1 — Main pUS21 cellular protein partners in T-REx-U2OS cells expressing pUS21-HA. [file mbio.00749-23-s0005.docx]

**TABLE S1.** Main pUS21 cellular protein partners in T-REx-U2OS cells expressing pUS21-HA as detected by mass spectrometry analysis

| **Statistical Accuracy^a^ Log(e)** | **Protein Name^b^** | **Gene** |
| --- | --- | --- |
| -30.8 | Talin-1 | TLN1 |
| -8.2 | UNC-13 homolog D | UNC13D |
| -7.4 | Tumor necrosis factor receptor superfamily member 10A | TNFRSF10A |
| -1.8 | Rab11 family-interacting protein 4 | RAB11FIP4 |
| -1.7 | Radical S-adenosyl methionine  domain-containing protein 2 | RSAD2 |
| -1.2 | DNA-binding protein SATB1 | SATB1 |

**^a^**Statistical accuracy is expressed as Log(e), indicating the expectation that any particular protein assignment was made at random (E-value). Assignments were made using the MS-BLAST algorithm for the peptide sequences detected.

**^b^**None of these proteins were detected in immunoprecipitates from uninduced TREx-U2OS-US21-HA cells.
